# Materia Medica, General Therapeutics and Pharmacy

**Published:** 1868-05

**Authors:** 


### Selections.

Materia Medica, General Therapeutics and Pharmacy.—*Bichloride of Methylene as a General Anæsthetic.*—We extract from an extremely interesting lecture by Dr. B. W. Richardson, published in the *Medical Times and Gazette* (Nov. 2, 1867,) the following particulars with regard to this new anæsthetic agent.

The following are its physical characteristics:

"The bichloride of methylene is a colorless fluid, having an odor much like the odor of chloroform. It is pleasant to inhale as vapor, and it produces very little irritation of the fauces and air-passages, It boils at 88° Fahr. Its sp. gr. is 1.344. The sp. gr. of its vapor is 2.937; it is, therefore, nearly three times heavier than air.

"That we may have before us all these facts, and that we may be able to compare and contrast the properties of bichloride of methylene with those of other anæsthetics, I have written a table which will explain itself:

| | Boiling point Fahr. | Sp. gr. Water 1,000 | Density of vapor. Air 1. |
|---|---|---|---|
| Chloride of methyl | 6° | ---- | 1.745 |
| Bichloride of methylene | 88 | 1.344 | 2.937 |
| Terchloride of formlye—chloroform | 142 | 1.495 | 4.122 |
| Tetrachloride of Carbon | 172 | 1.599 | 5.321 |
| Ether $C_2H_5O$ | 92 | 0.720 | 1.547 |
| Amalyne $C_5H_{10}$ | 96 | 0.659 | 2.419 |

"A glance at this table gives at once the physical positions, absolute and relative, of the bichloride. It boils at a lower point than any of the other anæsthetics — lower even than ether, and fifty-four degrees lower than chloroform. Its specific gravity, both as a liquid and a vapor, is lower than chloroform, but much higher than ether or amylene. From its position physically, it combines many of the properties of chloroform with those of ether, and these peculiarities must be remembered in its administration. From its easier evaporation, it requires more free administration than chloroform; and from its greater density of vapor, it requires less in quantity than ether.

"There is another physical difference between the bichloride and chloroform to which I would particularly invite your observation. If I take chloroform and diffuse the vapor of it through air in a bell jar thus, I find, when a taper alight is plunged into the jar, that the light is extinguished — in other words, the combustion is stopped. On further inquiry, I also find that the chloroform itself, though it has stopped the combustion, has itself undergone no obvious chemical change. We say, therefore, that the chloroform has acted by a catalytic process; it has stopped oxidation by its mere presence, without undergoing decomposition. I take next the bichloride of methylene, diffuse that in vapor through the jar, and plunge in the lighted taper. And now see the difference; the vapor burns in a brilliant flame, filling the jar. Here I have decomposed the substance; the carbon has been turned, by union with oxygen, into carbonic acid, and the hydrogen and the chlorine have been turned, by their new union, into hydrochloric acid. The proof of this latter fact concludes a singularly pretty experiment; I pour a few drops of strong ammonia into the jar in which the bichloride of methylene has been burned, and I produce a dense cloud of chloride of ammonium in white vapor, which pours out of the jar like water. I have been careful in showing these experiments with chloroform and bichloride of methylene, and the different behaviors of the latter in the presence of flame, because the experiments bear on one of the most able and ingenious theories ever put forward to explain the action of the anæsthetics on living organisms. Some of you will know that I refer to the theory of Dr. Snow. Snow, observing that the vapor of chloroform extinguished flame, as we have just seen, reasoned that, as it thus stopped the combustion of a taper, so by its catalytic action it stopped the combustion of the blood, from which arrest all the after phenomena of anæsthesia took their origin. 'I could demonstrate all the phenomena of anæsthesia on a farthing candle,' was one of his striking epigrams. But here we have a true anæsthetic, which will burn readily, giving brisk combustion. This fact, in so far as it goes, is not in accordance with the theory of my late distinguished friend, and in pointing out the fact I do no more in correction than I should for a favored theory of my own, or than he would, were he here to speak for himself.

"The bichloride of methyl mixes readily and well with absolute ether, and as the two fluids have nearly the same boiling point — four degrees of temperature being the extreme difference — when they are combined they form a compound which vaporizes evenly and equally. The difference in the specific gravities of the two vapors is the only objection to the combination. The bichloride further combines with chloroform in all proportions.

"One more physical matter in respect to the bichloride of methylene, and this part of the subject may be concluded. The fluids should have at all times a neutral re-action to test-paper. If it show any acidity, there is present a trace of hydrochloric acid, and the vapor, which under such circumstances would also contain the acid, would be irritating to the throat, and perhaps dangerous to life."

After learning by repeated experiments on inferior animals that it could be safely administered to them, Dr. R. inhaled it himself until it produced insensibility. "I found the vapor," he says, "very pleasant to breathe, and little irritating, while drowsiness came on and unconsciousness without any noise in the head or oppression. I recovered also as the animals seemed to recover — at once and completely. I felt, in fact, as though I had merely shut my eyes and had opened them again. In the mean time, however, I had performed certain acts of a motor kind unconsciously; for I inhaled the vapor in the laboratory, and there went to sleep, but I awoke in the yard adjoining. This was on September 28th. I inhaled on the occasion from a cup-shaped sponge. Since then, I have inhaled the vapor in smaller quantities from several instruments, was the effect of proving that there is little difference required for administration between the bichloride and chloroform."

Like all other general anæsthetics, the bichloride of methylene has power to destroy life. Its safety must, therefore, be accepted as relative rather than absolute. Dr. R. has tried to ascertain its relative value, and the result, he says, leads him "to hope that the balance of safety is on the side of the bichloride. Three observations bring me to this reasoning. First, I find that if two animals of the same age and kind, say pigeons, be placed in chambers of the same size, and exposed at the same temperature, and under other conditions the same, to equal values of chloroform, tetrachloride of carbon, and bichloride of methylene, the resistance to death will be as fourteen to five in favor of the bichloride of methylene against the tetrachloride of carbon, and as fourteen to nine against the chloroform.

"In the second place, when animals are exposed until they are killed by these vapors, there is a marked difference in the maintenance of muscular irritability. The tetrachloride of carbon destroys the muscular irritability first, the chloroform next, and the bichloride of methylene last, and this difference I have found so striking as to represent in one experiment a period of seven minutes for extinction of irritability by the tetrachloride, twenty-three minutes for the chloroform, and fifty-eight minutes for the bichloride of methylene. This distinction tests, I think, on difference in the amount of chlorine in the three substances, and I point out the fact not merely as showing the lower destructive power of the bichloride, but as affording a hope that in a case of accident from it the means resorted to for restoring animation would be more likely to succeed, the muscular power remaining more directly under the influence of excitants to renewed action, and for a long interval.

"Thirdly, the condition in which the lungs and heart are left after death from the bichloride is favorable."

The following are Dr. R.'s general conclusions in regard to the bichloride of methylene:

"1. It is an effective general anæsthetic, producing as deep insensibility as chloroform.

"2. In action it is rather more rapid than chloroform, but to develope effects more of it is required, in the proportion of six parts to four.

"3. It produces a less prolonged second degree of narcotism than other anæsthetics.

"4. When its effects are fully developed, the narcotism is very prolonged, and is re-produced with great ease.

"5. Its influence on the nervous centres is uniform, and it creates little, if any, disturbance or break of action between the respirating and circulating functions.

"6. Its final escape from the organism is rapid, so that the symptoms of recovery are sudden.

"7. In some cases it produces vomiting.

"8. When it kills it destroys by equally paralyzing the respirating and circulating mechanism.

"9. It interferes less with the muscular irritability than perhaps any other anæsthetic.

"10. It combines with ether and with chloroform in all proportions.

Dr. R., with characteristic candor and modesty, remarks: "I leave the bichloride of methylene with the profession for its observation and experience. I have proved the agent, by experiment on the lower animals, to be a good general anæsthetic. I have inhaled it myself with safety, and I have administered it to the human subject with success in the extremest operations for which general anæsthesia is demanded. Here, as an individual inquirer, I come back into the ranks and rejoin the rest of my brethren as an observer. Having no other ambition than that of being a physician in the widest sense, having even a painful aversion to specialty, and having no desire to press any subject unduly. I have produced this lecture as a contribution to pure science and nothing more, holding myself as free as any one else to condemn, improve, or approve, as future knowledge, framed and squared and fitted by wisdom, shall determine. When twenty thousand persons shall have slept away pain under the influence of 'Chloro-methyl,' as Mr. Spencer Wells has tersely named the bichloride of methylene, and those of them who have slept too deeply shall be counted as fewer than ten, an advance over chloroform will have been proved, but not sooner, nor with less of that tribulation through which we must ever attain to the good that is great and persistently beneficent."—*Am. Jour. Med. Sciences.*

